# A retrospective evaluation of activity of gemcitabine/platinum regimens in the treatment of recurrent ovarian cancer

**DOI:** 10.1186/s40661-017-0053-x

**Published:** 2017-11-14

**Authors:** Tran N. Le, Rachel E. Harvey, Christine K. Kim, Jubilee Brown, Robert L. Coleman, Judith A. Smith

**Affiliations:** 1 Department of Obstetrics, Gynecology, and Reproductive Sciences, UTHealth McGovern Medical School, 6431 Fannin Street, Rm. 3.152, Houston, TX 77030 USA; 2 MBS Pharmacy, Round Rock, TX USA; 3 University of Texas M.D. Anderson Cancer Center, Houston, TX USA; 4 Levine Cancer Institute, Carolinas HealthCare System, Charlotte, NC USA; 5 UTHealth-Memorial Hermann Cancer Center-TMC, Houston, TX USA

**Keywords:** Gemcitabine, Cisplatin, Carboplatin, Recurrent ovarian cancer, Efficacy, Toxicity

## Abstract

**Background:**

While many of these agents have been compared in prospective clinical trials, the gemcitabine/platinumbased regimens have not been compared in a prospective, randomized clinical trial. While bothgemcitabine/carboplatin and gemcitabine/cisplatin have a similar ORR in separate clinical trials, the tworegimens have never been directly been compared. With overlapping dose-limiting toxicity of thrombocytopenia, the gemcitabine/carboplatin regimen has been challenging to employ in the clinical setting in previously treated ovarian cancer patients and is often associated with treatment delays and/or dose reductions. Gemcitabine/cisplatin can also be a challenge due to its dose limiting neuropathy and renal toxicity, especially in previously treated patients. In the absence of any prospective, head to head comparison this retrospective study was embarked upon to compare the response rate and toxicity profiles of gemcitabine/cisplatin verses gemcitabine/carboplatin for the treatment of platinum-sensitive verses platinum-resistant recurrent ovarian cancer.

**Methods:**

This was a retrospective chart review study that identified patients that had received either gemcitabine/cisplatin or gemcitabine/carboplatin for treatment of recurrent ovarian cancer and compared documented hematological and non-hematological toxicity and response based on RECIST (v1.1). Data was evaluated based upon platinum sensitivity/resistance as well.

**Results:**

A total of 93 patients were identified that had received a gemcitabine/platinum regimen with 48 with recurrent ovarian cancer that were included in the study. There were 21 patients in the gemcitabine/cisplatin arm and 27 patients identified in the gemcitabine/carboplatin arm. Objective response rate (ORR) was greater in platinum-sensitive patients that received gemcitabine/carboplatin compared to gemcitabine/cisplatin (8 (67%) vs 2 (25%), *p* < 0.05). Conversely, ORR was greater in platinum-resistant patients treated with gemcitabine/cisplatin (4 (57%) vs 1 (25%), NS). Mean time to progression was greater in gemcitabine/cisplatin patients (7.2 vs 5.1 months, *p* < 0.03). Patients treated with gemcitabine/carboplatin discontinued due to toxicity at a greater rate (8 (33%) vs 5 (24%)). Specifically gemcitabine/carboplatin had a greater incidence (85%) of grade 2 or greater leukopenia, thrombocytopenia, and neutropenia compared to gemcitabine/cisplatin (19%) However, there was no significant difference in dose reductions, treatment delays, or granulocyte-colony stimulating factor (G-CSF) administration between regimens.

**Conclusions:**

Gemcitabine/cisplatin appears to have greater efficacy in platinum-resistant patients, while gemcitabine/carboplatin seems to have greater efficacy in platinum-sensitive patients. Overall, gemcitabine/carboplatin was associated with a greater incidence of myelosuppression and discontinuation due to toxicity. Similar to findings in endometrial cancer, gemcitabine/cisplatin may have benefit specifically in platinum-resistant ovarian cancer.

## Background

Ovarian cancer is one of the most common cancers of, and the leading cause of death from gynecologic cancers [1]. This cancer is often undiagnosed until its progression to stage III/IV, at which point the 5-year survival rate is less than 50% [2]. Most patients with advanced ovarian cancer develop recurrent disease which is usually resistant to many chemotherapeutic agents [2]. There is no official standard second-line treatment of recurrent ovarian cancer. The selection of chemotherapy for recurrent disease include is often based upon platinum-sensitivity, residual toxicity, and patient/physician preferences. It may include a combination regimen or single-agent regimen with various agents showing some benefit for treatment of recurrent ovarian cancer including: carboplatin, cisplatin, paclitaxel, pegylated liposomal doxorubicin, gemcitabine, topotecan, bevacizumab, or more recently one of the new PARP inhibitors.

While many of these agents have been compared in prospective clinical trials, the gemcitabine/platinum-based regimens have not been compared in a prospective, randomized clinical trial. Gemcitabine is a nucleoside analog which exerts its chemotherapeutic activity by incorporation into DNA causing apoptosis [3]. Gemcitabine possesses activity in ovarian cancer resistant to treatment with paclitaxel/carboplatin combination regimen, demonstrating synergy with cisplatin and a mild toxicity profile [3]. Neutropenia was the most commonly reported toxicity with gemcitabine [3]. Other reported toxicities include hematologic toxicity, flu-like symptoms, nausea, vomiting, and appetite suppression [3]. Cisplatin and carboplatin are platinum-based antineoplastic agents which bind to and cross-link DNA [4]. Both are used in combination with gemcitabine as treatment for recurrent ovarian cancer. Cisplatin-based chemotherapy has a high toxicity potential, most commonly causing nausea, vomiting, and other GI symptoms. [4] The primary dose-limiting toxicity of cisplatin is nephrotoxicity resulting in reduced renal perfusion and concentrating defect [5]. Carboplatin has lower reactivity and slower DNA binding kinetics in comparison to cisplatin [6]. Nausea and vomiting are less severe and easier to control in carboplatin compared to cisplatin [6]. Primary toxicities associated with carboplatin are myelosuppression and neutropenia [6].

Based on a previous randomized study by Pfisterer and colleagues, carboplatin in combination with gemcitabine demonstrates greater efficacy through improved overall response rate (ORR) and median progression free survival in comparison to carboplatin alone [7]. Gemcitabine/carboplatin also results in increased myelosuppression requiring increased supplementation with G-CSF, but sequelae such as febrile neutropenia and infections were uncommon. [7] While both gemcitabine/carboplatin and gemcitabine/cisplatin have a similar ORR in separate clinical trials [8, 9], the two regimens have never been directly been compared. With overlapping dose-limiting toxicity of thrombocytopenia, the gemcitabine/carboplatin regimen has been challenging to employ in the clinical setting in previously treated ovarian cancer patients and is often associated with treatment delays and/or dose reductions. Gemcitabine/cisplatin can also be a challenge due to its dose limiting neuropathy and renal toxicity, especially in previously treated patients. In the absence of any prospective, head to head comparison this retrospective study was embarked upon to compare the response rate and toxicity profiles of gemcitabine/cisplatin verses gemcitabine/carboplatin for the treatment of platinum-sensitive verses platinum-resistant recurrent ovarian cancer.

## Methods

### Patients

This retrospective review of medical records was performed on patients with recurrent ovarian cancer who completed treatment with either gemcitabine 1000 mg/m^2^ and cisplatin 40 mg/m^2^ biweekly regimen or gemcitabine 800 mg/m^2^ and carboplatin AUC 5 given once every 21 days at the University of Texas M.D. Anderson Cancer Center (UTMDACC) Gynecologic Oncology Center between 1 January 2002 to 30 September 2012. The protocol was reviewed and approved by the UTMDACC Institutional Review Board and granted a waiver of consent. Inclusion criteria included medical records of patients with recurrent ovarian cancer that received at least one cycle of gemcitabine/platinum based regimen. Medical records were excluded if patients were on concomitant biotherapy and/or other chemotherapy agents with gemcitabine/platinum regimen and patients with incomplete or restricted medical records.

### Data collection

Patient data extracted from medical records included: patient demographics, body mass index (BMI) at start of therapy, documented comorbidities, stage at diagnosis, tumor histology, tumor debulking history, chemotherapy history, baseline complete metabolic panel and complete blood count prior to each cycle of chemotherapy, number of dose reductions during treatment, number of treatment delays, complications of chemotherapy (use of rescue antiemetics, electrolyte replacement, IV hydration), and reason for discontinuation of regimen. Criteria established by Gordon and colleagues [10] were used to determine platinum-sensitive and –resistant disease. When patients stopped their respective regimen, response to treatment was evaluated by modified RECIST (version 1.1) based on measurable tumor progression [11].

### Statistical and data analysis

Within each gemcitabine/platinum group data were further sorted into platinum-sensitive and –resistant groups. The student t-test analysis was used to evaluate for statistically significant differences (*p* ≤ 0.05) between treatment groups in age, BMI, number of prior chemotherapy regimens, and number of cycles of gemcitabine/platinum completed. Time to progression was calculated as the interval between the start of the first gemcitabine/platinum cycle and the end of the last gemcitabine/platinum cycle; the end of the gemcitabine/cisplatin regimen was determined as 28 days after the last cycle start date, while the end of the gemcitabine/carboplatin regimen was determined as 21 days after the last cycle start date. The student t-test analysis was performed to evaluate for differences in time to progression, differences in dose reductions, treatment delays, and G-CSF administration between each treatment group with *p* values less than or equal to 0.05 considered significant. ORR was calculated on patients who completed treatment with the following responses considered an objective response: partial response to treatment, complete response, and stable disease. The chi-square test was performed to evaluate for differences in ORR. The incidence of grade 2 or greater leukopenia, thrombocytopenia, and neutropenia was measured and defined, respectively, as: white blood count < 3000/μL, platelet count < 75,000/μL, absolute neutrophil count < 1500/μL.

## Results

### Patient characteristics

A total of 93 charts were identified of patients that had received a gemcitabine/platinum regimen with 48 charts included from patients that had received gemcitabine/platinum for recurrent ovarian cancer from January 1st, 2002 to September 30th, 2012. Other than number of prior treatments, there were no statistical differences between patient demographics/characteristics as summarized in Table 1 by treatment group and by platinum sensitivity. Briefly there were 21 charts from patients that had received gemcitabine/cisplatin which included ten platinum-sensitive patients, ten platinum-resistant patients, and one patient whose platinum-sensitivity was unknown. There were 27 charts from patients that had received gemcitabine/carboplatin which included 18 platinum-sensitive patients, eight platinum-resistant patients, and one patient whose platinum-sensitivity was unknown. The majority of patients in both groups had serous tumors that were stage IIIC or IV whose tumor had been optimally debulked (<1 cm) prior to treatment. The gemcitabine/carboplatin group had a higher percentage of platinum-sensitive patients (66% vs 48%). There was no statistically significant difference between age or BMI in treatment groups. The mean number of prior chemotherapy regimens was significantly greater in the gemcitabine/cisplatin group, 3.5 ± 1.7 regimens (1–6, *p* = 0.003) compared to the gemcitabine/carboplatin group 2 ± 1.5 (0–6). When comparing treatment groups by platinum sensitivity, the gemcitabine/cisplatin platinum-sensitive subgroup had a greater number of mean prior chemotherapy regimens 3.2 ± 1.5 (1–5, *p* = 0.025) compared to the gemcitabine/carboplatin platinum-sensitive subgroup 1.8 ± 1.5 (0–6). There was no statistically significant difference in mean number of cycles of gemcitabine/platinum between groups.

**Table 1:** Summary of patient characteristics

Patient Demographics	Gem/Cis Overall	Gem/Carbo Overall	Gem-]/Cis Plt Sensitive	Gem/Carbo Plt Sensitive	Gem/Cis Plt Resistant	Gem/Carbo Plt Resistant
	*N* = 21	*N* = 27	*N = 10*	*N = 18*	*N = 10*	*N = 8*
Mean Age [# ± SD (Range)]	61.4 [±10.2(42–84)]	62.0 [+9.1(43–79)]	60.8 [+9.4(49–81)]	60.8 [+8.3(47–79)]	61.6 [+11.9(42–84)]	65.8 [+10.7(43–78)]
Race						
-White	16	20	7	14	8	5
-Hispanic	3	4	2	3	1	1
-African American	0	1	0	0	0	1
-Asian	0	1	0	1	0	0
-Unreported race	2	0	1	0	1	1
Mean # of Comorbidities	1.48	1.48	0.70	1.11	2.30	2.50
Tumor Histology						
-Serous	15	17	8	11	6	6
-Clear Cell	0	1	0	1	0	0
-Adenocarcinoma	2	2	2	0	0	1
-Mixed	1	3	0	3	1	1
-Unreported histology	3	3	0	3	3	0
Tumor Stage						
-I	0	1	0	0	0	1
-II	2	1	2	0	0	0
-III	12	17	5	11	6	5
-IV	3	3	2	3	1	0
-Unreported stage	4	6	1	4	3	2
Tumor Debulking						
-Optimal (<1 cm)	10	16	5	11	5	4
-Suboptimal	5	7	2	6	2	1
-Not a surgical candidate	0	1	0	0	0	1
-Unreported tumor debulking	6	3	3	1	3	2
Plt sens/resis/unkn	10/10/1	18/8/1	NA	NA	NA	NA
Mean # of prior regimens [# ± SD (Range)]	3.5 [+1.7(1–6)]	2 [+1.5(0–6)]	3.2 [+1.5(1–5)]	1.8 [+1.5(0–6)]	4 [+1.9(1–6)]	2.5 [+1.4(1–5)]
	*P* < 0.003		*P* < 0.025			
Mean # of cycles Gem/Plt [# ± SD (Range)]	6.2 [+3.5(1–12)]	5.44 [+2.5(2–12)]	6.7 [+3.5(2–12)]	5.3 [+2.2(2–9)]	5.2 [+3.0(1–9)]	6.3 [+3.0(3–12)]

*Abbreviations*: *Gem-Cis* Gemcitabine-cisplatin, *Gem-Carbo* Gemcitabine-carboplatin, *Plt* Platinum, *SD* Standard deviation, sens = sensitive; resis = resistant, unkn = unknown

### Efficacy

Table 2 summarizes response rates in each treatment group overall and by platinum-sensitivity. ORR was greater in the overall gemcitabine/carboplatin group (56% vs 38%). When comparing platinum-sensitive patients alone, the gemcitabine/carboplatin group again had a greater ORR (67% vs 25%, *p* < 0.05). However, when comparing platinum-resistant patients alone, the gemcitabine/cisplatin group had a greater ORR (57% vs 25%). Mean time to progression was significantly greater in the overall gemcitabine/cisplatin group 7.2 ± 2(3.2–9.7, *p* = 0.03) compared to the overall gemcitabine/carboplatin group 5.1 ± 1.7 (2.3–7.2). There was a higher percentage of patients who discontinued treatment due to toxicity in the overall gemcitabine/carboplatin group (33% vs 24%), in the platinum-sensitive gemcitabine/carboplatin subgroup (29% vs 20%), and in the platinum-resistant gemcitabine/carboplatin subgroup (75% vs 30%).

**Table 2:** Summary of response rates

Response Rates	Gemcitabine +Cisplatin	Gemcitabine + Carboplatin
*Overall*	*N* = 21	*N* = 24
Progression #(%)	10 (63)	7(44)
Complete response #(%)	2(13)	3(19)
Partial response #(%)	2(13)	3(19)
Stable disease #(%)	2(13)	3(19)
Discontinued due to toxicity #(%)	5(24)	8(33)
Objective response rate (PR + CR + SD) #(%)	6(38)	9(56)
Mean time to progression (months) [# ± SD (Range)] *p* = 0.03	7.2 [±2(3.2–9.7)]	5.1 [±1.7(2.3–7.2)]
Response Rates	Gemcitabine +Cisplatin	Gemcitabine + Carboplatin
*Platinum Sensitive*	*N = 10*	*N = 17*
Progression #(%)	6(75)	4(33)
Complete response #(%)	1(13)	3(25)
Partial response #(%)	0(0)	3(25)
Stable disease #(%)	1(13)	2(17)
Discontinued due to toxicity #(%)	2(20)	5(29)
Objective response rate (PR + CR + SD) #(%) *p* = 0.05	2(25)	8(67)
Mean time to progression (months) [# ± SD (Range)]	7.2 [±2.5(3.2–9.5)]	5.1 [±1.8(2.3–7.2)]
Response Rates	Gemcitabine +Cisplatin	Gemcitabine + Carboplatin
*Platinum Resistant*	*N = 10*	*N = 7*
Progression #(%)	3(43)	3(75)
Complete response #(%)	1(14)	0(0)
Partial response #(%)	2(29)	0(0)
Stable disease #(%)	1(14)	1(25)
Discontinued due to toxicity #(%)	3(30)	3(75)
Objective response rate (PR + CR + SD) #(%)	4(57)	1(25)
Mean time to progression (months) [# ± SD (Range)]	7.2 [±1.6(5.7–9.7)]	5.1 [±1.8(2.5–6.8)]

*Abbreviations*: *SD* Standard deviation, *PR* Partial response, *CR* Complete response, *SD* Stable disease

### Toxicities

Table 3 summarizes toxicity profiles of each treatment group. Use of rescue antiemetics was greater in the gemcitabine/cisplatin group compared to the gemcitabine/carboplatin group (29% vs 4%). Use of electrolyte replacement was greater in the gemcitabine/carboplatin group (33% vs 24%). Change in renal function from baseline occurred more often in the gemcitabine/cisplatin group (19% vs 4%). Change in liver function from baseline occurred more often in the overall gemcitabine/carboplatin group (22% vs 10%). There was no statistically significant difference in dose reductions or treatment delays in either treatment group. Mean rate of G-CSF administration per patient was greater in the gemcitabine/carboplatin group 4.2 ± 3.2 (0–9) compared to the gemcitabine/cisplatin group 2.9 ± 3.3 (0–9), however the difference was not statistically significant. In the gemcitabine/carboplatin group there was a greater rate of grade 2 or higher leukopenia (37% vs 0%), thrombocytopenia (7% vs 0%), and neutropenia (41% vs 19%). Of the 13 patients that discontinued due to toxicity, eight patients were from the gemcitabine/carboplatin group; five of these eight patients also had grade 2 or greater or myelosuppression.

**Table 3:** Summary of toxicity profiles

Toxicity Profiles	Gemcitabine +Cisplatin	Gemcitabine + Carboplatin
*Overall*	*N* = 21	*N* = 27
Use of rescue antiemetics #(%)	6(29)	1(4)
Use of electrolyte replacement #(%)	5(24)	9(33)
Use of additional IV hydration	7(33)	8(30)
Change in renal function from baseline #(%)	4(19)	1(4)
Change in liver function from baseline #(%)	2(10)	6(22)
Mean dose reductions per patient [# + SD (Range)]	1.9 [+0.7(1–3)]	1.9 [+0.7(1–3)]
Mean treatment delays per patient[# + SD (Range)]	0.8 [+0.9(0–3)]	0.7 [+1(0–4)]
Mean rate of G-CSF administration per patient [# + SD (Range)]	2.9 [+3.3(0–9)]	4.2 [+3.2(0–9)]
Leukopenia, Grade 2 or greater #(%)	0	10(37)
Thrombocytopenia, Grade 2 or greater #(%)	0	2(7)
Neutropenia, Grade 2 or greater #(%)	4(19)	11(41)

*Abbreviation*: *SD* Standard deviation

## Discussion

The results of this study comparing gemcitabine/carboplatin and gemcitabine/cisplatin regimens show that gemcitabine/carboplatin has greater efficacy in platinum-sensitive patients, but gemcitabine/cisplatin demonstrates greater efficacy in platinum-resistant patients. Toxicity profiles demonstrate decreased use of rescue antiemetics but greater myelosuppression with gemcitabine/carboplatin.

No previous studies have compared gemcitabine/cisplatin and gemcitabine/carboplatin in the treatment of recurrent ovarian cancer. However, the controversy of which platinum to used in combination with paclitaxel three randomized studies have compared efficacy and toxicity of paclitaxel/carboplatin with paclitaxel/cisplatin in the first-line treatment of advanced ovarian cancer. In a phase III study, Nejit and colleagues randomized 208 patients with advanced ovarian cancer to paclitaxel/cisplatin or paclitaxel/carboplatin to compare toxicity profiles [11]. Overall, paclitaxel/carboplatin was less toxic with fewer patients discontinuing due to toxicity, less nausea and vomiting, and less peripheral neurotoxicity. However, paclitaxel/carboplatin resulted in a greater incidence of granulocytopenia and thrombocytopenia. In another phase III trial, Ozols and colleagues randomized 792 patients with optimally resected stage III ovarian cancer to receive paclitaxel/cisplatin or paclitaxel/carboplatin [12]. Again, paclitaxel/cisplatin demonstrated increased gastrointestinal, renal, and metabolic toxicity while paclitaxel/carboplatin had a greater incidence of grade 2 or greater thrombocytopenia. No significant difference was observed in the regimens’ efficacy measured by median progression-free survival, overall survival, relative risk of progression, and relative risk of death. Similarly, Du Bois and colleagues also concluded paclitaxel/carboplatin and paclitaxel/cisplatin are comparable in terms of efficacy, but paclitaxel/carboplatin led to a higher frequency of hematologic toxicity [13].

Similar to the three randomized studies comparing paclitaxel/cisplatin with paclitaxel/carboplatin, this study indicates gemcitabine/carboplatin results in greater myelosuppression than gemcitabine/cisplatin. Grade 2 or greater leukopenia, thrombocytopenia, and neutropenia occurred at a markedly greater rate in the gemcitabine/carboplatin group compared to the gemcitabine/cisplatin group. Although there was no significant difference in dose reductions, treatment delays, or rate of G-CSF administration per patient, the gemcitabine/carboplatin displayed a greater rate of discontinuation due to toxicity. Due to the retrospective nature of this study, the reason for discontinuation due to toxicity was not specified for all patients. However, most of the gemcitabine/carboplatin patients who discontinued due to toxicity also demonstrated grade 2 or greater leukopenia, neutropenia or thrombocytopenia. This is in agreement with studies showing greater myelosuppression with gemcitabine/carboplatin than carboplatin alone, as well as with paclitaxel/carboplatin compared to paclitaxel/cisplatin [7, 11–13]. To further emphasize this observation of greater myelosuppression in the gemcitabine/carboplatin group, it is important to note that the gemcitabine/cisplatin group actually had a significantly greater mean number of prior chemotherapy regimens that would increase the likelihood of being more susceptible to the development of myelosuppression.

Other aspects of the toxicity profile of gemcitabine/carboplatin compared to gemcitabine/cisplatin in this study are similar to previous studies [11, 12]. In particular, a greater rate of use of rescue antiemetics and incidence of change in renal function was observed more often in the gemcitabine/cisplatin group. The greater incidence of change in renal function in the gemcitabine/cisplatin patients was expected since carboplatin dosing is based on individualized renal function and *cisplatin* is not.

In contrast to prior studies on paclitaxel/cisplatin and paclitaxel/carboplatin that found no difference in efficacy between regimens [12, 13], this retrospective study observed a greater ORR in the gemcitabine/carboplatin group overall. When the response rates were evaluated based on platinum sensitivity, gemcitabine/carboplatin had a greater ORR in platinum-sensitive patients compared to gemcitabine/cisplatin. However, this study observed that gemcitabine/cisplatin had a greater ORR in platinum-resistant patients than those that received gemcitabine/carboplatin. Previous studies have demonstrated gemcitabine/cisplatin activity in platinum-resistant patients [14] and clinically it is predominantly administered to patients with platinum-resistant disease [15, 16]. A larger is study is needed to ascertain whether gemcitabine/cisplatin is more effective in platinum-resistant patients compared to gemcitabine/carboplatin.

Furthermore, the mean time to progression was statistically significantly greater in gemcitabine/cisplatin patients regardless of platinum -sensitivity even though tumor stage, histology, and rate of optimal debulking were similar between the treatment groups. Interestingly, gemcitabine/cisplatin patients had a statistically significantly greater mean number of prior regimens, which would theoretically cause greater rates of drug resistance and decrease mean time to progression. However, Smith and colleagues have previously demonstrated in-vitro the increase cytotoxicity observed with gemcitabine/cisplatin was attributed to gemcitabine modulation of cisplatin-resistance in a panel of human endometrial cancer cell lines [17, 18]. These findings translated to clinical practice as demonstrated in follow up phase II clinical study by Brown and colleagues that observed an significantly improved PFS and objective response in platinum-resistant endometrial carcinoma [19]. In addition, Smith and colleagues went onto confirm combination as well as sequential treatment with gemcitabine with cisplatin demonstrated a greater improvement in growth inhibitory activity in both the chemosensitive and chemoresistant ovarian cancer cell lines which was attributed to modulation of the steroid xenobiotic receptor/multi-drug resistance (SXR/MDR) pathway [20]. In this study gemcitabine/cisplatin was found to be more active than gemcitabine/carboplatin in the platinum-resistant patients perhaps this is because gemcitabine and cisplatin are given on the same day twice a cycle which allows for optimal time for the gemcitabine modulation of the platinum-resistance pathways leading to improved sensitivity to the cisplatin activity. In the gemcitabine/carboplatin regimen both drugs are given on day one only then day 8 is the gemcitabine alone which has less cytotoxicity activity by itself in recurrent, platinum-resistant ovarian cancer. A definitive reason is currently unknown.

Since this was a retrospective study design, the study does have limitations that could not be controlled. First, the overall small sample size for the study. There are multiple options for chemotherapy treatment options for recurrent ovarian cancer, hence this limited sample size. Patients that did not have confirmed diagnosis of ovarian cancer were excluded to attempt to limit sources of variability in response rates. The patients on the gemcitabine/cisplatin arm did have slightly higher number of prior treatments overall as well as when evaluated by platinum-sensitivity too. It likely contributed to the lower overall response rate and lower response in the “platinum-sensitive” patients too. Conversely, despite having a higher number of prior treatments, the gemcitabine/cisplatin regimen had better response in those patients with documented platinum-resistance. This observation supports the hypothesis that gemcitabine modulated multi-drug resistance pathways. In the absence of confirmatory data, selection of which gemcitabine/platinum regimen should be based on the common principles of selection of chemotherapy for recurrent ovarian cancer including patient convenience and residual toxicity. In those patients with known platinum resistance, the current pre-clinical and clinical data suggests gemcitabine/cisplatin appears to have more likelihood of achieving a response.

## Conclusion

This study is the first study to directly compare gemcitabine/carboplatin to gemcitabine/cisplatin in patients with recurrent ovarian cancer. Overall the incidence of toxicity was similar between the two regimens and consistent with previous studies of carboplatin, gemcitabine/carboplatin leads to greater myelosuppression than gemcitabine/cisplatin. It appears that efficacy of gemcitabine/carboplatin seems to have greater efficacy in platinum-sensitive patients. Similar to findings in platinum-resistant endometrial cancer and well as recent clinical trials in platinum-resistant ovarian cancer, gemcitabine/cisplatin appears to have greater efficacy in platinum-resistant patients [14, 15, 18]. Preliminary in vitro data suggests gemcitabine may have a role in modulation of the SXR/MDR which would improve sensitivity of multiple chemotherapy agents in used for the treatment recurrent drug resistant tumors [19]. Based on this current study, additional research efforts should focus on way optimize the role of gemcitabine/cisplatin for treatment of platinum-resistant ovarian cancer.

## Abbreviations

BMI: Body Mass Index
G-CSF: Granulocyte-colony stimulating factor
NS: Not significant
ORR: Objective response rate
SXR/MDR: Steroid xenobiotic receptor/multi-drug resistance
UTMDACC: University of Texas M.D. Anderson Cancer Center
